# Speed synchronization, physical workload and match-to-match performance variation of elite football players

**DOI:** 10.1371/journal.pone.0200019

**Published:** 2018-07-24

**Authors:** Bruno Gonçalves, Diogo Coutinho, Bruno Travassos, Hugo Folgado, Pedro Caixinha, Jaime Sampaio

**Affiliations:** 1 University of Trás-os-Montes and Alto Douro, Vila Real, Portugal; 2 Research Center in Sports Sciences, Health Sciences and Human Development, CIDESD, CreativeLab Research Community, Vila Real, Portugal; 3 University of Beira Interior, Covilhã, Portugal; 4 University of Évora, Évora, Portugal; Sao Paulo State University - UNESP, BRAZIL

## Abstract

This study aimed to: (i) examine whether the speed synchronization and physical performance of an elite football team changed between the first and the second half, using match time blocks of 15-min, and (ii) explore the match-to-match variation of players’ speed synchronization performance. Twenty-eight outfield elite footballers participated in 51 official matches. Positional data were gathered and used to calculate the total distance covered as a physical workload indicator. For all the outfield teammate dyad combinations (45 pairs), it was processed the percentage of time that players’ speed was synchronized during walking, jogging and running using relative phase (Hilbert Transform). Also, the match-to-match variation of the players’ speed synchronization, expressed in coefficient of variation was computed. The differences in the total distance covered from all players within the different match’s time block periods revealed a moderate decrease in the distance covered in the last 15-min of the match compared to the first 15-min (-6.5; ±1.07%, most likely: change in means with 95% confidence limits). However, when compared the last minutes from both halves a small increase was observed (2.7; ±1.2%, likely) from first to second half. The synchronization of the players’ speed displacements revealed small to moderate decreases in the % of synchronization in the second half periods for the jogging and running speed, while the opposite was found for the walking speed (~13 to 24% more, most likely). The playing position analysis for the walking zone showed similar trends between the groups, with small to moderate higher values in the second half, with the exception of [30’-45’] vs [75’-90’] in the midfielder’s dyads and in [15’-30’] vs [60’-75’] match periods for forwards. Similar trend was found during the running speed, in which small to moderate higher synchronization was found during the first half periods, with the exception of [15’-30’] vs [60’-75’] and [30’-45’] vs [75’-90’] in midfielder’s dyads. Regarding to the match-to-match variation of the players’ speed synchronization, overall results showed small to moderate increases in coefficient of variation during jogging and running displacements from the beginning to the end of the match (32.1; ±13.2% increase in jogging and 26.2; ±10.5% in running, both comparisons most likely). The higher distance covered during most of the first half periods and the higher dyadic synchronization at high speeds might have limited players’ performance in the second half. In addition, the decrease trend in speed synchronization during the second half periods might have resulted from accumulated muscular and mental fatigue towards the match. Within, the match-to-match variation in tactical-related variables increased across the match duration, with especial focus in the midfielder dyads. Dyadic speed synchronization might provide relevant information concerning the individual and collective performance.

## Introduction

The analysis of team sports performance requires a multidimensional approach that helps to capture the adaptive behaviour of players and teams [1, 2]. Football is a complex team sport, in which the players’ performance derived from their interaction with the surrounding context information that sustain the emergence of the players’ physical, technical and tactical actions [3–6]. According to that, during the last years, it has been highlighting that a more comprehensive interpretation of players’ performance might emerge if different performance perspectives, such as tactical and physical indicators, are considered together [3, 4, 7]. However, most of previous studies have been focused in only one dimension of performance (i.e. physical, technical or tactical actions), or approach them independently, neglecting the mutual influence between them. For example, time-motion analysis has been extensively used to analyse and characterize the competitive workload demands of football. Generally, it was showed that players cover between 10 to 13 km during an official match [8, 9]. However, only around 10% from this total distance covered represents the hight intensity movements, since most of the movement activities are performed at walking and running speed zones [8, 9]. Furthermore, players’ physical performance seems to be different between match halves, with declines being reported during the second half [10–13]. Accordingly, these changes are suggested to be related with high values of distance covered at jogging and running during the first half [14], which may limit the players’ performance during the second half [11].

While the division of the activity demands from the first to the second half allowed a better understanding on how players’ effort changes across a competitive match, analysing players physical performance during shorter periods might provide additional information. In this sense, Mohr et al. [12] reported decrements of 15% to 45% in the distance covered in high-intensity running in the last 15-min compared with the first half 15-min match periods. Similarly, Carling et al. [15] analysed the total distance covered and the high-speed distance covered during 15-min match intervals in midfielders and found higher values in these variables during the first 15-min of the match compared with the last 15-min (75-min to 90-min).

Research has also demonstrated that the activity demands are dependent on specific players’ position [11, 14, 16]. Midfielders have been shown to cover more distance than defenders or forwards [11, 16]. Furthermore, when considering the distance covered at high speeds, both forwards and midfielders revealed higher distance covered than defenders [10, 11]. However, such demands are not constant for the entire match and can change according to the periods of the match in analysis. Indeed, research has shown decrements in the total distance covered for the midfielders and defenders when compared the first minutes of the second half with the same period in the first half [14]. Also, both forwards and midfielders shown decrements in high-intensity running mainly in the final part of the second half [14].

Clearly, the analysis of players’ physical performance during competitive matches has been considerably investigated [8, 12, 15]. In turn, the analysis of tactical performance or the integration of physical and tactical behaviour to understand the emergent team performance is still scarce [5]. For example, the analysis of tactical behaviour in team sports have been assessed through relative phase processing technique to understand the spatial-temporal coordination between players direction displacements during football competitive matches as proposed by McGarry et al. [17]. Following previous works in Basketball [18] and Futsal [19], some authors quantified the time that players spent synchronized in longitudinal and lateral directions as a tactical performance indicator [3, 4, 20]. Considering that players seems to coordinate their actions with the aim of achieve a common goal in a shared surrounding [5, 21], higher time spent synchronized has been linked with high tactical performances [7]. Overall, tactical analysis revealed that players exhibit higher interpersonal coordination tendencies when facing strong oppositions [3]. In turn, lower team movement synchronization has been found when teams faced congested periods, and mental fatigue has been pointed as a possibly reason for this decrease [20]. However, one limitation from this approach is that it capture the players’ movement synchronization divided into longitudinal and lateral movements, not considering the compounded direction of players’ movements. Thus, an integrated variable that captures players’ synchronization should be used to improve the understanding of players’ tactical performance. In this regard, the use of speed continuously captures the adjustments of players on the field and allows to capture the rhythmic of move and the level of coordination between them. In fact, high intensity displacements have been linked with goal-scoring opportunities [22] and with actions that could lead to break the symmetry with the opponents defence [23]. Thus, a better understanding on how players coordinate their actions might emerge if the analysis of movement synchronization considers the speed of players’ displacements, however, no study to date has addressed this issue. Also, the analysis of movement synchronization between players and teams over the match time allows to capture the dynamics of tactical behaviour of players and teams in a reliable way. For instance, the analysis of teams’ behaviour across 15-min time block periods during one match, revealed differences in teams dispersion across periods and more regular patterns towards the end of the match [24].

Another important issue that has been gathering research interest over the last years is the concept of the match-to-match variation of the player’s performance [25–30]. In general, it was revealed that match-to-match variations occurs especially in technical parameters and in high-speed running workloads. According to such results, it seems very important to improve current knowledge by addressing match-to-match variations in tactical-related variables. The outcomes may address reference values that allow coaches to better understand the adaptability of the own team according to match environment. Accordingly, performance variability can be seen as advantageous, because it reflects adjustments of repeated behaviours to the dynamic environment [31]. Functional variations might reflect the behaviour flexibility when facing environmental boundary conditions [32] as occur over the match.

Based on previous assumptions, it is clear that performance analysis is a multidimensional approach. Thus, since physical behaviour reflects an exploratory behaviour of players to perform, research that crosses tactical and physical variables are required to provide more accurate and reliable information regarding players performance variations during matches [1, 5, 33]. Also, no study has addressed how players’ positional role and match time periods constraint their players’ tactical and physical performance during competitive matches. In addition, while the performance variabily may reflect players’ ability to adapt to the environmental information [31], in fact, the match-to-match variation in tactical-realted variables is an unknown topic. Thus, this study aimed to: (i) examine whether the tactical and physical performances of an elite football team varied between the first and the second half using 15-min match time blocks and (ii) explore the match-to-match variation of players tactical performances. In line with previous research it was expected that the physical performance decreased from the first minutes to the last minutes of the match with differences according to the players’ role. Also, it was expected to observe different trends on tactical behaviour and match-to-match performance variation for defenders, midfielders and forwards.

## Materials and methods

### Participants

Twenty-eight male outfield professional football players (age: 24.7±4.7 y; height: 178.2±6.2 cm; weight: 72.9±6.7 kg; professional playing experience 6.5±4.7 y) participated in 51 official matches. For each match, the analysis have only considered the players who were part of the starting line-up and performed the entire match duration. The club technical staff and the players provided a written and informed consent to participate in this study after a detailed explanation about the aims and risks involved in the investigation. The study protocol was approved and followed the guidelines stated by the Ethics Committee of the of University of Trás-os-Montes and Alto Douro, based ate Vila Real (Portugal) and conformed to the recommendations of the Declaration of Helsinki.

### Data collection and processing

The positional data from each outfield player were tracked and collected using the Match Analysis Camera System®. The system records the precise location of all 22 players on the field at subsecond frequency. The total distance covered was computed as physical workload indicator. Taking into consideration the all-possible intra-team dyads formed by the outfield teammates (45 dyads), it was processed the frequency of near-in-phase synchronization from the players’ speed displacements (expressed in % of time). After, this variable was processed (and the % of time calculated) based on the mean speed from dyads according to following intensity zones (adapted from Folgado et al. [3]): walking (0.0 to 3.5 km.h^-1^), jogging (3.6 to 14.3 km.h^-1^) and running (≥14.4 km.h^-1^). The considered variables were processed for all intra-team dyads and it was only considered the defender, midfielders and forward dyads. Also, to evaluate the changes associated with evolving time across the match, six match time blocks of 15-mins were considered and analysed as following periods [24, 30]: [0’-15’]; [15’-30’]; [30’-45’]; [45’-60’]; [60’-75’]; [75’-90’]). The extra time in both halves was not accounted to keep consistent the matches duration.

The Hilbert Transform [34] was used to compute the relative phase of the time series corresponding to speed displacements of all dyads. Near-in-phase synchronization (i.e. % of time spent between -30° and 30° of relative phase) was used to access players’ interpersonal speed coordination. This method has been recently proposed to better inform on the dynamics of coordination between dyads in effective performance contexts [3, 35, 36], however no study has applied it to the players’ speed displacements time series.

The within-dyads match-to-match variation was expressed by the coefficient of variation (CV) of each match case [26, 27, 37]. The differences of variation of match performance between players’ speed displacement dyads were compared according to the match time periods and playing positions. In order to calculate the within-dyads CV, only the dyads who performed at least two entire matches were selected.

### Statistical analysis

Magnitude-based inferences and precision of estimation were used to analyse the data [38, 39]. Prior to the comparisons, all processed variables were log-transformed to reduce the non-uniformity of error. Descriptive analysis were graphically represented using individual case values and mean±standard deviations for all variables (the presented mean is the back-transformed mean of the log transform). Differences in within-match time periods ([0’-15’] vs [45’-60’]; [15’-30’] vs [60’-75’]; [30’-45’] vs [75’-90’]; and [0’-15’] vs [75’-90’]) were expressed in percentage units with 95% confidence limits. The threshold for a change to be considered practically important (the smallest worthwhile difference) was 0.2 x between standard deviation. Uncertainty in the true effects of the conditions were evaluated based on non-clinical inferences. The following magnitudes of clear effects were considered: <0.5%, most unlikely; 0.5–5%, very unlikely; 5–25%, unlikely; 25 to 75%, possibly; 75% to 95% likely; 95% to 99%, very likely; >99% most likely [40]. Also, the within-match time periods comparisons were assessed via standardized mean differences and respective 95% confidence intervals. Thresholds for effect sizes statistics were 0.2, trivial; 0.6, small; 1.2, moderate; 2.0, large; and >2.0, very large [40]. All statistical computations were processed with a specific post-only crossover spreadsheet for all players and when considering only defenders, midfielders and forwards [41].

## Results

**Fig 1:**
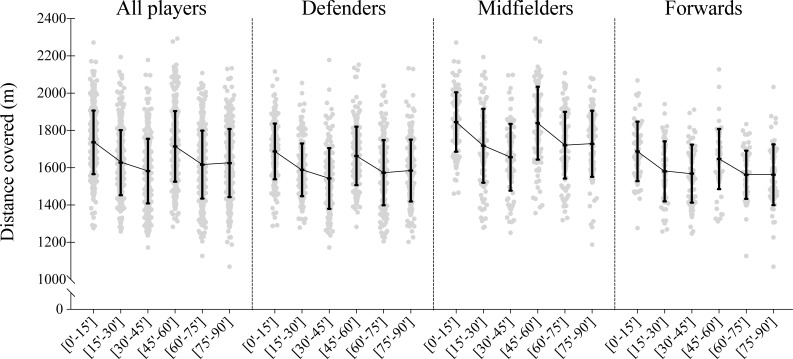
Descriptive values for players’ distance covered according to the match period and playing positions. Each dot represents an individual value and the black error bars indicate mean±standard deviation.

**Fig 2:**
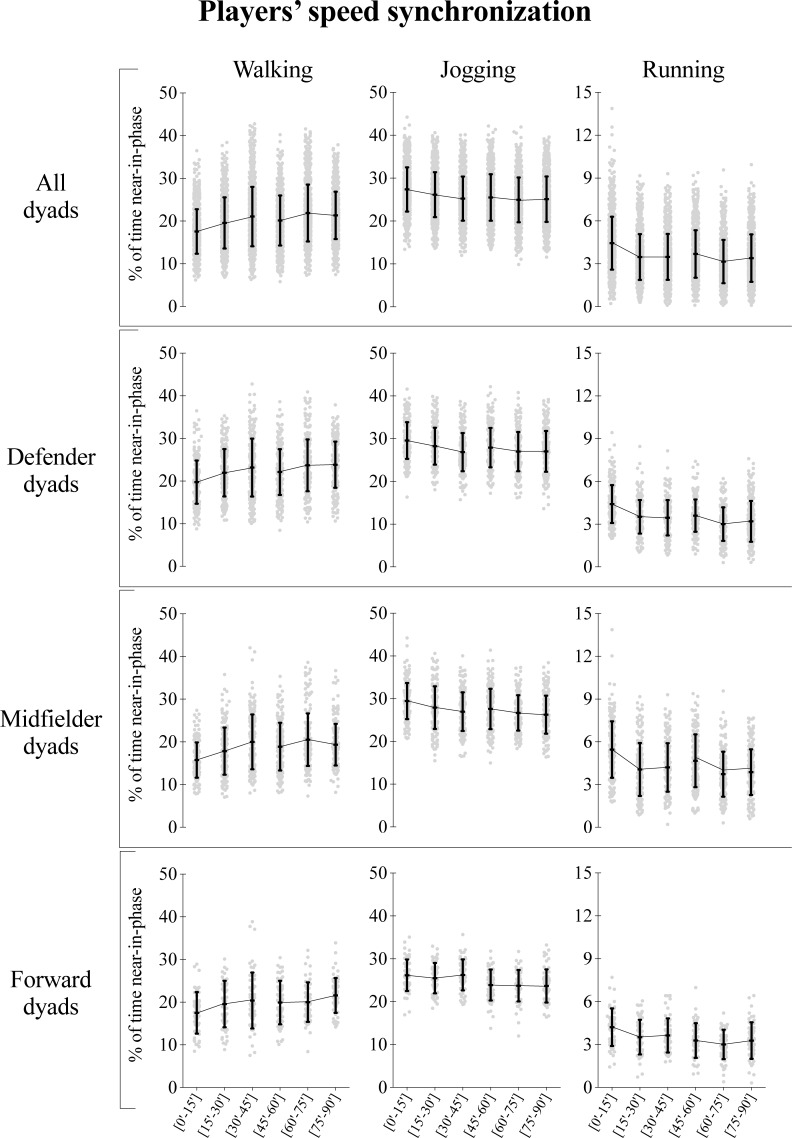
Descriptive values for players’ speed synchronization according to the match period and playing positions while walking, jogging and running. Each dot represents an intra-team dyad value and the black error bars indicate mean±standard deviation.

**Fig 3:**
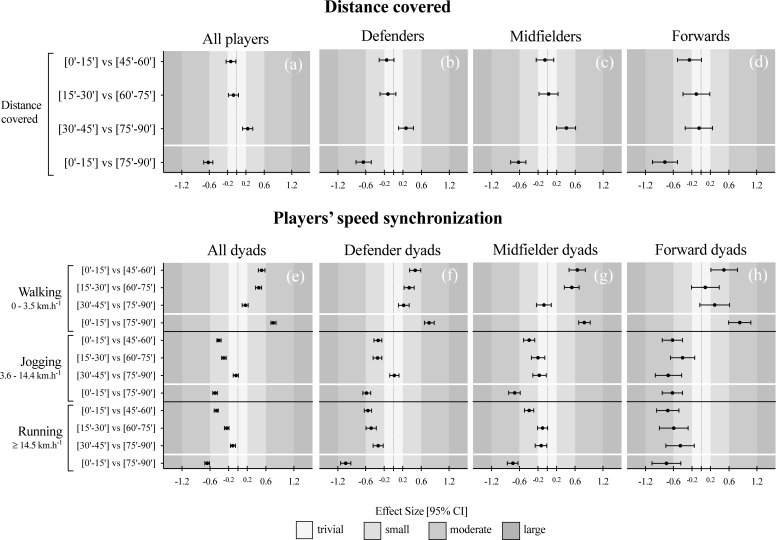
Standardized (Cohen) differences in players’ distance covered and speed synchronization at different intensity zones and according to different match periods and playing positions (left panel: a, e–all dyads; left central panel: b, f–defender dyads; right central panel: c, g–midfielder dyads; right panel: d, h–forward dyads). Error bars indicate uncertainty in the true mean changes with 95% confidence intervals.

Descriptive analysis can be observed in both Fig 1 and Fig 2 for distance covered and speed displacements synchronization of the teammate’s dyads, respectively. The corresponding differences from all players within the different match’s time block periods (i.e. [0’-15’] vs [45’-60’]; [15’-30’] vs [60’-75’]; [30’-45’] vs [75’-90’]; and [0’-15’] vs [75’-90’]) are shown in Table 1. Finally, the Fig 3 presents the standardized (Cohen) differences for all considered previous mentioned comparisons.

**Table 1:** Inferences for the within-match periods comparisons according to playing positions. Change in means and uncertainty in the true differences for players’ distance covered and speed synchronization dyads.

Variables	Change in means (%; ±95%CL)			
	Uncertainty in the true differences			
	[0’-15’] vs [45’-60’]	[15’-30’] vs [60’-75’]	[30’-45’] vs [75’-90’]	[0’-15’] vs [75’-90’]
**Distance covered (m)**				
**All players**	-1.4; ±1.1	-0.7; ±1.2	2.7; ±1.2	-6.5; ±1.0
	likely tri	very likely tri	likely ↑	most likely ↓
**Defenders**	-1.5; ±1.6	-1.2; ±1.6	2.7; ±1.6	-6.2; ±1.5
	possibly ↓	likely tri	likely ↑	most likely ↓
**Midfielders**	-0.5; ±2.0	0.3; ±2.2	4.4; ±2.3	-6.5; ±1.7
	likely tri	likely tri	very likely ↑	most likely ↓
**Forwards**	-2.5; ±2.5	-1.0; ±2.9	-0.4; ±3.0	-7.5; ±2.5
	possibly ↓	possibly ↓	unclear	most likely ↓
**Players’ speed synchronization (% of time near-in-phase)**				
**All dyads**				
Walking (0–3.5 km.h^-1^)	15.5; ±2.2	13.4; ±2.1	4.6; ±1.9	24.0; ±1.8
	most likely ↑	most likely ↑	likely tri	most likely ↑
Jogging (3.6–14.4 km.h^-1^)	-7.4; ±0.8	-5.5; ±0.9	-0.8; ±1.0	-8.8; ±0.8
	most likely ↓	most likely ↓	most likely tri	most likely ↓
Running (≥14.4 km.h^-1^)	-20.7; ±1.7	-11.3; ±2.3	-5.1; ±2.8	-28.1; ±1.9
	most likely ↓	likely ↓	most likely tri	most likely ↓
**Defender dyads**				
Walking (0–3.5 km.h^-1^)	13.0; ±3.5	9.4; ±3.2	5.9; ±3.2	22.4; ±3.3
	most likely ↑	very likely ↑	possibly ↑	most likely ↑
Jogging (3.6–14.4 km.h^-1^)	-5.2; ±1.3	-5.3; ±1.4	0.4; ±1.6	-8.8; ±1.3
	most likely ↓	most likely ↓	most likely tri	most likely ↓
Running (≥14.4 km.h^-1^)	-19.5; ±2.5	-17.4; ±3.5	-12.3; ±4.0	-33.6; ±3.0
	most likely ↓	most likely ↓	very likely ↓	most likely ↓
**Midfielder dyads**				
Walking (0–3.5 km.h^-1^)	13.0; ±3.5	9.4; ±3.2	5.9; ±3.2	22.4; ±3.3
	most likely ↑	very likely ↑	possibly ↑	most likely ↑
Jogging (3.6–14.4 km.h^-1^)	-6.3; ±1.9	-3.2; ±2.3	-2.7; ±2.3	-11.1; ±1.9
	most likely ↓	possibly ↓	possibly ↓	most likely ↓
Running (≥14.4 km.h^-1^)	-16.9; ±3.9	-4.5; ±5.1	-6.2; ±5.6	-29.9; ±3.9
	most likely ↓	very likely tri	likely tri	most likely ↓
**Forward dyads**				
Walking (0–3.5 km.h^-1^)	14.2; ±8.8	2.4; ±8.4	8.2; ±9.4	25.1; ±8.2
	very likely ↑	unclear	possibly ↑	most likely ↑
Jogging (3.6–14.4 km.h^-1^)	-9.3; ±3.1	-6.1; ±3.8	-10.5; ±4.0	-9.3; ±3.1
	most likely ↓	likely ↓	most likely ↓	most likely ↓
Running (≥14.4 km.h^-1^)	-25.7; ±7.3	-21.6; ±9.9	-16.9; ±10.5	-26.6; ±9.3
	most likely ↓	very likely ↓	likely ↓	most likely ↓

Symbols and abbreviations: ↓ = decrease; ↑ = increase; tri = trivial; ‘ = minutes; m = meters.

The results from all players revealed a moderate decrease in the distance covered in the last minutes of the match, i.e. [75’-90’], compared to the first minutes of the match, i.e., [0’-15’] (change in means; ±95% confidence limits: -6.5; ±1.1%, most likely). However, when compared the last minutes from both halves ([30’-45’] vs [75’-90’]), a small increase was observed (2.7; ±1.2%, likely) from first to second half. Similar results were found when accounting for the different playing position analyses, as both the defenders and midfielders showed a moderate decrease in the distance covered in the [75’-90’] compared to the [0’-15’] (~6 to 7% less, most likely) and a small increase from the [30’-45’] to [75’-90’] (~3 to 4% more, likely). Although the forwards showed a moderate decrease in the distance covered comparing the [0’-15’] vs [75’-90’] (-7.5; ±2.5%, most likely), unclear/trivial effects were found in all the other match periods comparisons (see Fig 1 and Table 1 for complement statistical information).

**Fig 4:**
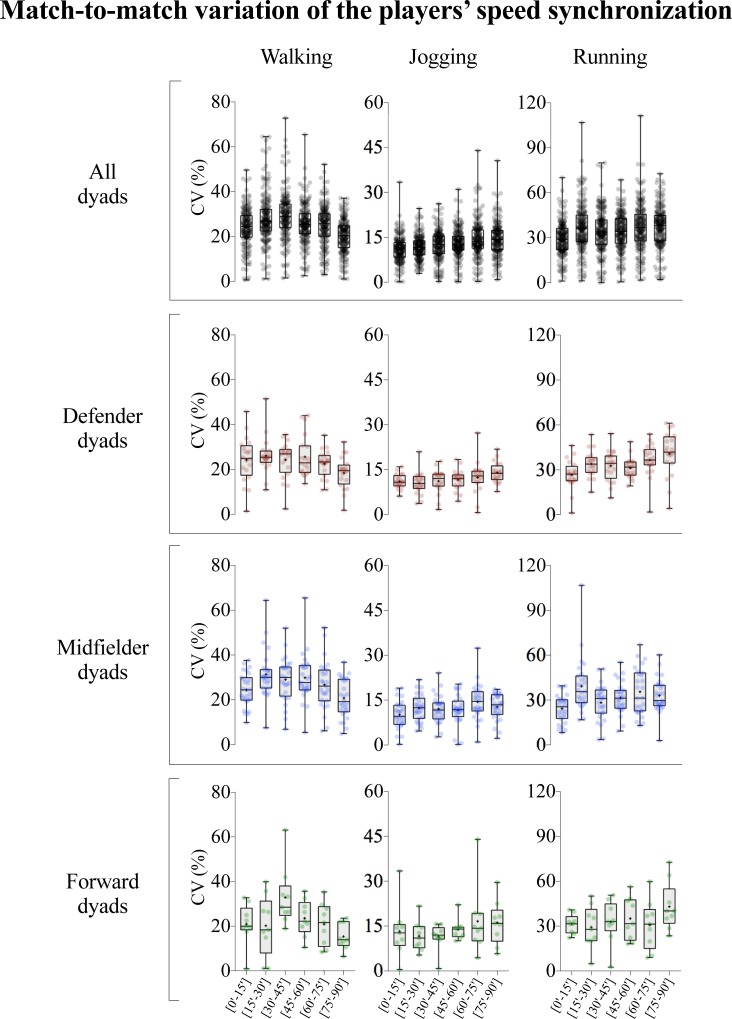
Match-to-match variation of the players’ speed synchronization. Each dot represents the coefficient of variation (CV) for each intra-team dyad value. The whiskers connect all points, from the minimum to the maximum; + represents the mean; and the box middle solid line represents the median.

The data from all teammate’s dyads (Fig 3E) revealed a small to moderate decrease in the % of synchronization in the second half periods for the jogging and running speed, while the opposite was found for the walking speed with small to moderate increase (~13 to 24% more, most likely). The playing positions analysis (Fig 2 and Fig 4F, 4G and 4H) for the walking zone showed similar trends between the groups, with small to moderate higher values in the second half, with the exception of [30’-45’] vs [75’-90’] in the midfielder dyads and in [15’-30’] vs [60’-75’] periods in forwards. In contrast, small to moderate increases in the synchronization were found during the first half periods for the jogging speed, apart from the [15’-30’] vs [60’-75’] in midfielder dyads, and in the [30’-45’] vs [75’-90’] in both the defender and midfielder dyads. Similar trend was found during the running speed, in which small to moderate increases in synchronization was found during the first half periods, with the exception of [15’-30’] vs [60’-75’] and in last 15-min of each half for midfielders (see Table 1, Figs 2 and 3 for complement statistical information).

The descriptive and inferential results for the match-to-match variation of the players’ speed synchronization, expressed in CV, according to match period and playing positions can be observed in both Fig 4 and Table 2, respectively. Considering all dyads, the CV increased across first half and decrease till the end of the match during walking displacements. However, during jogging and running displacements, the match-to-match variation showed small to moderate increases from the beginning to the end of the match ([0’-15’] vs [75’-90’]: 32.1; ±13.2% increase in jogging and 26.2; ±10.5% in running, both comparisons most likely). The defender dyads showed unclear trend for walking values and a moderate increase in the last period of the match when considering the jogging and running displacements. The midfielder dyads showed small to moderate decreases in the walking zones during the last period of second half, compared to the first, and small to moderate increases in jogging and running zones in same comparisons. In addition, the forward dyads showed unclear results for the majority of the comparisons, however a moderate decrease was identified in [30’-45’] vs [75’-90’] match-to-match variation during walking displacements (-53.8; ±18.3, most likely) and a small increase in [0’-15’] vs [75’-90’] during running (31.6; ±40.5, likely).

**Table 2:** Inferences for the match-to-match variation of the players’ speed synchronization, expressed in coefficient of variation, according to match period and playing positions. Change in means, uncertainty in the true differences and standardized differences.

Variables	Change in means (%; ±95%CL)			
	Uncertainty in the true differences			
	Standardized differences (Cohen; ±95%CL)			
	[0’-15’] vs [45’-60’]	[15’-30’] vs [60’-75’]	[30’-45’] vs [75’-90’]	[0’-15’] vs [75’-90’]
**Players’ speed synchronization: match-to-match variation**				
**All dyads**				
Walking (0–3.5 km.h^-1^)	10.1; ±10.8	-10.8; ±7.9	-31.8; ±6.3	-15.4; ±8.5
	possibly ↑	possibly ↓	most likely ↓	likely ↓
	0.19; ±0.2	-0.23; ±0.18	-0.77; ±0.18	-0.34; ±0.2
Jogging (3.6–14.4 km.h^-1^)	18.6; ±13.0	17.2; ±11.2	20.5; ±12.5	32.1; ±13.2
	likely ↑	likely ↑	likely ↑	most likely ↑
	0.31; ±0.20	0.29; ±0.17	0.34; ±0.19	0.51; ±0.18
Running (≥14.4 km.h^-1^)	16.0; ±11.5	2.3; ±10.6	13.1; ±11.9	26.2; ±10.5
	possibly ↑	very likely tri	possibly ↑	most likely ↑
	0.26; ±0.17	0.04; ±0.18	0.21; ±0.18	0.40; ±0.14
**Defender dyads**				
Walking (0–3.5 km.h^-1^)	17.2; ±49.2	-13.4; ±21.8	-25.2; ±33.2	-21.2; ±42.6
	unclear	unclear	unclear	unclear
	0.30; ±0.76	-0.27; ±0.46	-0.54; ±0.80	-0.44; ±0.96
Jogging (3.6–14.4 km.h^-1^)	2.4; ±18.9	6.1; ±50.7	34.6; ±39.6	25.8; ±22.9
	unclear	unclear	likely ↑	likely ↑
	0.05; ±0.38	0.12; ±0.95	0.61; ±0.60	0.47; ±0.37
Running (≥14.4 km.h^-1^)	32.4; ±52.2	-1.5; ±37.2	17.6; ±39.1	56.9; ±32.1
	likely ↑	unclear	unclear	most likely ↑
	0.51; ±0.69	-0.03; ±0.66	0.29; ±0.59	0.81; ±0.37
**Midfielder dyads**				
Walking (0–3.5 km.h^-1^)	20.1; ±27.7	-18.2; ±15.1	-30.3; ±12.8	-19; ±17.5
	likely ↑	likely ↓	most likely ↓	likely ↓
	0.41; ±0.52	-0.45; ±0.41	-0.82; ±0.41	-0.48; ±0.48
Jogging (3.6–14.4 km.h^-1^)	7.2; ±62.9	11.0; ±26.0	5.3; ±21.3	39.7; ±42.8
	unclear	possibly ↑	unclear	likely ↑
	0.10; ±0.79	0.15; ±0.33	0.07; ±0.28	0.47; ±0.43
Running (≥14.4 km.h^-1^)	33.0; ±29.0	-11.5; ±18.6	22.5; ±42.9	35.6; ±28.8
	very likely ↑	possibly ↓	unclear	very likely ↑
	0.57; ±0.43	-0.24; ±0.42	0.40; ±0.68	0.61; ±0.42
**Forward dyads**				
Walking (0–3.5 km.h^-1^)	39.2; ±126.8	54.2; ±192.4	-53.8; ±18.3	-10.3; ±93.2
	unclear	unclear	most likely ↓	unclear
	0.37; ±0.91	0.48; ±1.17	-0.86; ±0.43	-0.12; ±1.02
Jogging (3.6–14.4 km.h^-1^)	45.4; ±158.9	30.1; ±112.4	57.1; ±115.7	55.7; ±138.7
	unclear	unclear	unclear	unclear
	0.47; ±1.18	0.33; ±0.97	0.56; ±0.85	0.55; ±1
Running (≥14.4 km.h^-1^)	5.0; ±37.5	5.2; ±60.0	53.0; ±147.1	31.6; ±40.5
	unclear	unclear	unclear	likely ↑
	0.07; ±0.54	0.08; ±0.83	0.65; ±1.31	0.42; ±0.46

Symbols and abbreviations: ↓ = decrease; ↑ = increase; tri = trivial; ‘ = minutes.

## Discussion

This study aimed to (i) examine whether the players’ speed synchronization and physical performances of an elite football team varied between the first and the second half using 15-min match time block periods. Also, (ii) it explored the match-to-match variation of players’ speed synchronization performance. The findings revealed higher dyadic synchronization at higher speeds (jogging and running) during the first half and higher dyadic synchronization at low speed (walking) during the second half. The specific positioning analysis showed less effects between halves for the midfielders, while the forwards showed a clear decrease in dyadic synchronization at high speeds during the second half. Also, the match-to-match variation on tactical performance increased across the match duration, with especial focus to the midfielders dyads.

During the match, the competing teams develop tactical adjustments through dynamic spatial-temporal relations within teammates and opponents with the aim of stablish space dominance and numerical superiority [42]. However, such adjustments on space dominance and numerical superiority vary according to changes on the game and strategical shared information between players. For example, at the beginning of the match, players probably use their teammates positioning to regulate their behaviour, as result of the strategical aspects trained for the match preparation. During the match progression, as the local information about the opponents’ performance builds up, players may adapt their behaviour accordingly. In fact, the results found in the match-to-match variation of players’ speed synchronization seems to support these findings. That is, the team in analysis started the match with higher regularity (less variability) in their behaviours, however, as the match progressed it can be depicted an increase in the variability in speed displacements synchronization between teammates. Accordingly, the movement variability has been related with a characteristic of dynamical adaptive systems and also of the best teams [17], whereas it may reveal the ability to adapt to the environmental requirements. In fact, these results suggest that teammates change the team’s interactions over the natural course of the matches through the recognition of the spatial-temporal relations and the collective possibilities for action that sustain their functional behavior [24].

The analysis of players’ movement synchronization has been used as a performance indicator that aims to assess the tactical behaviour of players from the same team [3, 7, 20, 35], and it has been suggested that higher time spent synchronized may reflect better tactical performances, considering the overall extent of the match [7, 20, 43]. While the spatial proximity seems to contribute to higher level of movement synchronization, in addition, the speed of displacement seems also to be a key factor [3, 44]. In fact, it has been shown that high speed movements behaviours are linked with decisive actions in football, such as disrupting the opposing team [23] and goal scoring situations [22]. Thus, the movement speed seems to reflect the way how players explore the performance context to adjust their behaviour and to ensure functional spatial-temporal relations within teammates and opponents according to the different match moments and intensity demands.

The results from our study revealed that different dyadic speed displacements’ synchronization was found when considering different speed categories between halves. For instance, the first half presented higher speed synchronization at jogging and running speeds, while in turn, the second half showed higher speed synchronization at walking. Similarly, previous studies have shown decrements in the physical performance during the second half when compared with the first, possibly as result of the accumulated muscular fatigue [10, 12, 13]. Accordingly, the distance covered at high intensity during the second half seems to be influenced by the activity of the first half [14], and therefore, the lower values in dyadic synchronization found during the second half may be related with the higher dyadic synchronization at higher speeds during the first half. It can be identified a decreasing trend in the speed displacements synchronization from the [0’-15’] vs [45’-60’], to the [15’-30’] vs [60’-75’] towards the [30’-45’] vs [75’-90]. In addition to the muscular fatigue, another possible reason for the results may be related with mental fatigue. That is, the ability to perform movements of high intensity and maintain dyadic synchronization require perceptual and decision-making skills, such as attention [45], accuracy and speed of decision making [46], that have been found to be diminished during periods of mental fatigue. For example, lower movement synchronization has been identified during moments of mental fatigue in football [43]. As so, the lower speed synchronization found at higher speeds towards the matches’ halves might suggest that players experience periods of mental fatigue during the match, mainly during the second half, with consequences on their movement synchronization. The results from the physical variables seems to support these findings, as in general higher distance was covered in the [75’-90’] period compared to the [30’-45’] period. These outcomes may indicate that players have to increase their distance covered in an attempt to correct and adjust their positioning due to lower interpersonal movement speed coordination [7].

The specific playing position analysis showed similar dyadic speed synchronization during different intensity zones. In this sense, all considered dyads showed higher synchronization at high speeds during the first half compared to the second half. The opposite was observed for low intensity displacements. Thus, higher speed synchronization was found during the beginning of the match with a trend to decrease over time. These results may be related with the team strategy or even match events. In fact, different team behaviours have been shown to emerge when considering different time block periods [24] and were related with match critical moments [47]. Therefore, the higher dyadic speed synchronization, in line with the higher distance covered found in this period, may suggests specific team behaviour (e.g. applying a high pressure on the opponent). In the same line of reasoning, some other authors showed that the match-to-match variation in total high-speed running declines across halves [30]. However, the same authors arise some doubts on the appropriateness of general measures of high-speed activity for determining variability in an elite soccer team. Accordingly, the results from this study may contribute to a more holistic overview about how the players’ performance change in a match-to-match basis.

The midfielders showed trivial effects between time periods in the dyadic synchronization at high speeds between the [15’-30’] vs [60’-75’] and between the [30’-45’] vs [75’-90’]. This outcome seems to reinforce previous findings that the first match period may be related with specific and strategical movement behaviours. Furthermore, it may be realted with the key role of these players, which have the main responsibility of linking the defensive and offensive sectors, by controlling the space and maintaining a more regular coordination with them [4]. Also, the midfields have been found to evidence high scores in decision-making and positioning than the other sectors [48], and thus, it may attenuate the detrimental effects in movement synchronization in other playing positions towards the end of the match. The increase of the speed synchronization CV according to match-to-match variation during jogging and running over the course of match (and based on unclear differences in defender and forward dyads), emphasizes the specificity of this playing position role according to the match contextual requirement. The forwards clearly showed lower results of dyadic synchronization during the second half at higher speed categories. A determined movement speed is required for the players to achieve collective behaviours [44], and previous reports shown that there is a decrease in the distance covered while jogging and running during the second half, mainly in jogging speed for forwards [11].

This study points out new insights about changes in players’ dyadic speed synchronization according to temporal changes during football competitive matches. Common movement synchronization analysis has been done using the players’ positional data, which allows to understand the time that players spent synchronized in the longitudinal and lateral directions. However, players may also be synchronized during other type of movements (e.g. diagonal movements), and therefore using the players dyadic speed synchronization may complement the information derived from positional data, and provide an update understanding on how players coordinate their actions at different speeds. Nevertheless, some limitations should be acknowledged such as it was only considered the players who played the entire match, and therefore, substitute players might have influenced the behaviour of the whole team.

## Conclusions

The assessment of dyadic synchronization during competitive matches and how it changes from to match-to-match seems to provide relevant information regarding on how players couple their movements according to different speed categories. Accordingly, this study found higher dyadic speed synchronization during the first half at high speed categories (jogging and running), while during the second half the players showed higher dyadic synchronization while walking. The decrement in speed synchronization towards the match as well as their increase in match-to-match variation (from [0’-15’] vs [45’-60’] to [30’-45’] vs [75’-90]) might suggest that players experience mental fatigue, as they are exposed to highly variable contextual situations with consequences on their physical and tactical performances. Despite higher distance covered, less half effects were found on the midfielders, mainly between [15’-30’] vs [60’-75’] and [30’-45’] vs [75’-90], possibly due to their key role in linking all sectors, as well as their possibly higher ability of decision making and positioning skills. Overall, the decrements of dyadic synchronization found during the second half, mainly for forwards, may indicate that coaches should prepare physical and mental fatiguing practice tasks to increase players ability to adapt and perform under these scenarios. Also, coaches may expect an increase variation in their players’ performances over the match and therefore, the training situation may benefit if some of these concerns become trainable. That is, the training exercises should expose players to different match conditions that promote adaptability on the behaviour of players and teams over time. Also, specific training tasks can, and should, be developed under fatigue situations, both mental and physical, where the contextual information (e.g. winning/losing, unbalance, etc.) change continuously.

## References

[1] Carling C, Wright C, Nelson L, Bradley P. Comment on ‘Performance analysis in football: A critical review and implications for future research’. J Sports Sci. 2013:1–6. 10.1080/02640414.2013.807352 23886412

[2] Gonçalves B, Coutinho D, Santos S, Lago-Penas C, Jimenez S, Sampaio J. Exploring Team Passing Networks and Player Movement Dynamics in Youth Association Football. PLoS One. 2017;12(1):e0171156 10.1371/journal.pone.0171156 28141823 PMC5283742

[3] Folgado H, Duarte R, Fernandes O, Sampaio J. Competing with Lower Level Opponents Decreases Intra-Team Movement Synchronization and Time-Motion Demands during Pre-Season Soccer Matches. PLoS ONE. 2014;9(5):e97145 10.1371/journal.pone.0097145 24817186 PMC4016249

[4] Gonçalves B, Figueira B, Macãs V, Sampaio J. Effect of player position on movement behaviour, physical and physiological performances during an 11-a-side football game. J Sports Sci. 2014;32(2):191–9. 10.1080/02640414.2013.816761 24016056

[5] Memmert D, Lemmink KA, Sampaio J. Current Approaches to Tactical Performance Analyses in Soccer Using Position Data. Sports Med (Auckland, NZ). 2016. 10.1007/s40279-016-0562-5 27251334

[6] Ric A, Torrents C, Gonçalves B, Torres-Ronda L, Sampaio J, Hristovski R. Dynamics of tactical behaviour in association football when manipulating players' space of interaction. PLoS ONE. 2017;12(7):e0180773 10.1371/journal.pone.0180773 28708868 PMC5510826

[7] Folgado H, Gonçalves B, Sampaio J. Positional synchronization affects physical and physiological responses to preseason in professional football (soccer). Res Sports Med. 2018;26(1):51–63. 10.1080/15438627.2017.1393754 29058465

[8] Bangsbo J, Mohr M, Krustrup P. Physical and metabolic demands of training and match-play in the elite football player. J Sports Sci. 2006;24(7):665–74. 10.1080/02640410500482529 16766496

[9] Bradley PS, Carling C, Gomez Diaz A, Hood P, Barnes C, Ade J, et al Match performance and physical capacity of players in the top three competitive standards of English professional soccer. Hum Mov Sci. 2013;32(4):808–21. 10.1016/j.humov.2013.06.002 23978417

[10] Di Salvo V, Gregson W, Atkinson G, Tordoff P, Drust B. Analysis of high intensity activity in Premier League soccer. Int J Sports Med. 2009;30(3):205–12. 10.1055/s-0028-1105950 19214939

[11] Lago C, Rey E, Lago-Ballesteros J, Casais L, Domínguez E. Analys of work-rate in soccer according to playing position. Int J Perf Anal Spor. 2009;9:218–27.

[12] Mohr M, Krustrup P, Bangsbo J. Match performance of high-standard soccer players with special reference to development of fatigue. J Sports Sci. 2003;21(7):519–28. 10.1080/0264041031000071182 12848386

[13] Bradley PS, Di Mascio M, Peart D, Olsen P, Sheldon B. High-Intensity Activity Profiles of Elite Soccer Players at Different Performance Levels. J Strength Cond Res. 2010;24(9):2343–51. 10.1519/JSC.0b013e3181aeb1b3 19918194

[14] Bradley PS, Noakes TD. Match running performance fluctuations in elite soccer: Indicative of fatigue, pacing or situational influences? J Sports Sci. 2013:1–12. 10.1080/02640414.2012.720701 23808376

[15] Carling C, Dupont G. Are declines in physical performance associated with a reduction in skill-related performance during professional soccer match-play? J Sports Sci. 2011;29(1):63–71. 10.1080/02640414.2010.521945 21077004

[16] Di Salvo V, Baron R, Tschan H, Montero FJ, Bachl N, Pigozzi F. Performance characteristics according to playing position in elite soccer. Int J Sports Med. 2007;28(3):222–7. 10.1055/s-2006-924294 17024626

[17] McGarry T, Anderson DI, Wallace SA, Hughes MD, Franks IM. Sport competition as a dynamical self-organizing system. J Sports Sci. 2002;20(10):771–81. 10.1080/026404102320675620 12363294

[18] Bourbousson J, Sève C, McGarry T. Space-time coordination patterns in basketball: Part 1—Intra- and inter-couplings amongst player dyads. J Sports Sci. 2010;28 (3):339–47. 10.1080/02640410903503632 20131146

[19] Travassos B, Araújo D, Duarte R, McGarry T. Spatiotemporal coordination patterns in futsal (indoor football) are guided by informational game constraints. Hum Mov Sci. 2012;31(4):932–45. 10.1016/j.humov.2011.10.004 22672740

[20] Folgado H, Duarte R, Marques P, Sampaio J. The effects of congested fixtures period on tactical and physical performance in elite football. J Sports Sci. 2015;33(12):1238–47. 10.1080/02640414.2015.1022576 25765524

[21] Silva P, Travassos B, Vilar L, Aguiar P, Davids K, Araujo D, et al Numerical relations and skill level constrain co-adaptive behaviors of agents in sports teams. PLoS One. 2014;9(9):e107112 10.1371/journal.pone.0107112 25191870 PMC4156427

[22] Faude O, Koch T, Meyer T. Straight sprinting is the most frequent action in goal situations in professional football. J Sports Sci. 2012;30(7):625–31. 10.1080/02640414.2012.665940 22394328

[23] Carling C, Bloomfield J, Nelsen L, Reilly T. The Role of Motion Analysis in Elite Soccer Contemporary Performance Measurement Techniques and Work Rate Data. Sports Med. 2008;38(10):839–62. 18803436 10.2165/00007256-200838100-00004

[24] Duarte R, Araujo D, Folgado H, Esteves P, Marques P, Davids K. Capturing complex, non-linear team behaviours during competitive football performance. J Syst Sci Complex. 2013;26(1):62–72.

[25] Gregson W, Drust B, Atkinson G, Salvo VD. Match-to-match variability of high-speed activities in premier league soccer. Int J Sports Med. 2010;31(4):237–42. 10.1055/s-0030-1247546 20157871

[26] Kempton T, Sullivan C, Bilsborough JC, Cordy J, Coutts AJ. Match-to-match variation in physical activity and technical skill measures in professional Australian Football. J Sci Med Sport. 2015;18(1):109–13. 10.1016/j.jsams.2013.12.006 24444753

[27] Liu H, Gomez MA, Gonçalves B, Sampaio J. Technical performance and match-to-match variation in elite football teams. J Sports Sci. 2016;34(6):509–18. 10.1080/02640414.2015.1117121 26613399

[28] Trewin J, Meylan C, Varley MC, Cronin J. The match-to-match variation of match-running in elite female soccer. J Sci Med Sport.21(2):196–201. 10.1016/j.jsams.2017.05.009 28595867

[29] Rampinini E, Coutts AJ, Castagna C, Sassi R, Impellizzeri FM. Variation in top level soccer match performance. Int J Sports Med. 2007;28(12):1018–24. 10.1055/s-2007-965158 17497575

[30] Carling C, Bradley P, McCall A, Dupont G. Match-to-match variability in high-speed running activity in a professional soccer team. J Sports Sci. 2016;34(24):2215–23. 10.1080/02640414.2016.1176228 27144879

[31] Stergiou N, Yu Y, Kyvelidou A. A perspective on human movement variability with applications in infancy motor development. Kinesiol Rev. 2013;2: 93–102.

[32] Stergiou N. Nonlinear Analysis for Human Movement Variability: Taylor & Francis, Taylor & Francis, a CRC title, part of the Taylor & Francis imprint, a member of the Taylor & Francis Group, the academic division of T&F Informa plc; 2016.

[33] Travassos B, Davids K, Araujo D, Esteves PT. Performance analysis in team sports: Advances from an Ecological Dynamics approach. Int J Perf Anal Spor. 2013;13(1):83–95.

[34] Palut Y, Zanone PG. A dynamical analysis of tennis: Concepts and data. J Sports Sci. 2005;23(10):1021–32. 10.1080/02640410400021682 16194979

[35] Fernandes O, Folgado H, Duarte R, Malta P. Validation of the tool for applied and contextual time-series observation. Int J Sport Psychol. 2010;41:63–4.

[36] Gonçalves B, Esteves P, Folgado H, Ric A, Torrents C, Sampaio J. Effects of Pitch Area-Restrictions on Tactical Behavior, Physical, and Physiological Performances in Soccer Large-Sided Games. J Strength Cond Res. 2017;31(9):2398–408. 10.1519/JSC.0000000000001700 27806007

[37] Spencer M, Losnegard T, Hallen J, Hopkins WG. Variability and Predictability of Performance Times of Elite Cross-Country Skiers. Int J Sport Physiol. 2014;9(1):5–11. 10.1123/ijspp.2012-0382 23799826

[38] Batterham AM, Hopkins WG. Making Meaningful Inferences About Magnitudes. Int J Sport Physiol. 2006;1(1):50–7. 19114737

[39] Buchheit M. The Numbers Will Love You Back in Return-I Promise. Int J Sport Physiol. 2016;11(4):551–4. 10.1123/IJSPP.2016-0214 27164726

[40] Hopkins WG, Marshall SW, Batterham AM, Hanin J. Progressive Statistics for Studies in Sports Medicine and Exercise Science. Med Sci Sport Exer. 2009;41(1):3–12. 10.1249/MSS.0b013e31818cb278 19092709

[41] Hopkins WG. Spreadsheets for Analysis of Controlled Trials, Crossovers and Time Series. Sportscience [Internet]. 2017; 2017(21):[1–4 pp.]. Available from: sportsci.org/2017/wghxls.htm.

[42] Weston M, Batterham AM, Castagna C, Portas MD, Barnes C, Harley J, et al Reduction in Physical Match Performance at the Start of the Second Half in Elite Soccer. Int J Sport Physiol. 2011;6(2):174–82. 10.1123/ijspp.6.2.174 21725103

[43] Coutinho D, Gonçalves B, Travassos B, Wong DP, Coutts AJ, Sampaio JE. Mental Fatigue and Spatial References Impair Soccer Players' Physical and Tactical Performances. Front Psychol. 2017;8:1645 10.3389/fpsyg.2017.01645 28983273 PMC5613114

[44] Gonçalves B, Folgado H, Coutinho D, Marcelino R, Wong Del P, Leite N, et al Changes in effective playing space when considering sub-groups of 3 to 10 players in professional soccer matches. J Hum Kinet. 2018. 10.1515/hukin-2017-0166 PMC6006547 29922386

[45] Boksem MA, Meijman TF, Lorist MM. Effects of mental fatigue on attention: an ERP study. Brain Res Cogn Brain Res. 2005;25(1):107–16. 10.1016/j.cogbrainres.2005.04.011 15913965

[46] Smith MR, Zeuwts L, Lenoir M, Hens N, De Jong LM, Coutts AJ. Mental fatigue impairs soccer-specific decision-making skill. J Sports Sci. 2016;34(14):1297–304. 10.1080/02640414.2016.1156241 26949830

[47] Lago-Penas C, Dellal A. Ball Possession Strategies in Elite Soccer According to the Evolution of the Match-Score: the Influence of Situational Variables. J Hum Kinet. 2010;25:93–100.

[48] Kannekens R, Elferink-Gemser MT, Visscher C. Positioning and deciding: key factors for talent development in soccer. Scand J Med Sci Spor. 2011;21(6):846–52. 10.1111/j.1600-0838.2010.01104.x 22126715

